# Genotypes and Haplotypes in the *AXIN2* and *TCF7L2* Genes are Associated With Susceptibility and With Clinicopathological Characteristics in Breast Cancer Patients

**DOI:** 10.3389/bjbs.2021.10211

**Published:** 2022-01-25

**Authors:** M. A. Rosales-Reynoso, V. Rosas-Enríquez, A. M. Saucedo-Sariñana, M. Pérez-Coria, M. P. Gallegos-Arreola, E. Salas-González, P. Barros-Núñez, C. I. Juárez-Vázquez, S. E. Flores-Martínez, J. Sánchez-Corona

**Affiliations:** ^1^ Division of Molecular Medicine, Center for Western Biomedical Research (CIBO), Guadalajara, Mexico; ^2^ Service of Medical Oncology, High Specialty Medical Unit, Hospital of Gynecology and Obstetrics, Guadalajara, Mexico; ^3^ Division of Genetics, Center for Western Biomedical Research (CIBO), Guadalajara, Mexico; ^4^ Unit of Follow-up Research of Metabolic Diseases, UMAE Pediatrics, Centro Médico Nacional de Occidente (CMNO), Instituto Mexicano del Seguro Social (IMSS), Guadalajara, Mexico; ^5^ Academic Directorate Devices and Systems I, Faculty of Medicine, Dean of Health Sciences, Autonomous University of Guadalajara (UAG), Guadalajara, Mexico

**Keywords:** breast cancer, *AXIN2*, *TCF7L2*, genotypes, haplotypes, susceptibility

## Abstract

**Background:** Breast cancer is a multifactorial disease whose genetic susceptibility is related to polymorphic variants of cell proliferation and migration pathways. Variants in *AXIN2* and *TCF7L2* in the Wnt-β catenin pathway have been associated with different types of cancer; however, little is known about its role in breast cancer. This study tests the hypothesis of links between *AXIN2* rs1133683 and rs2240308, and *TCF7L2* rs7903146 and rs12255372 variants in breast cancer.

**Methods:** Peripheral blood samples were obtained from 404 women (202 patients and 202 control females). The polymerase chain reaction-restriction fragment length polymorphism (PCR-RFLP) methodology was used to identify the gene variants.

**Results:** The *AXIN2* rs2240308 (C > T), and *TCF7L2* rs7903146 (C > T) and rs12255372 (G > T) variants were associated with breast cancer and with age, TNM stage, and histologic-molecular subtype (*p* = 0.001). Likewise, the haplotype T-T in the *TCF7L2* gene (rs7903146-rs12253372) was significantly related with breast cancer (OR = 2.66, 95%, CI = 1.64–4.30, *p* = 0.001).

**Conclusion:** Our data show a link between *AXIN2* rs2240308 and *TCF7L2* rs7903146 and rs12255372 variants in breast cancer, and speculate this may be important in pathogenesis.

## Introduction

The World Health Organization highlight that breast cancer constitutes a significant worldwide health problem and is the leading cause of death among women (1). The estimated number of women with breast cancer in 2018 was 651,000, accounting for more than a third of worldwide cancer cases (2). However, survival rates among breast cancer patients in the developed world are much higher than those in less developed countries (3). Breast cancer is a complex disease influenced by a variety of factors, including the low education level, smoking, the environment, family history, obesity, hormones, and the immune system (4). Genetic risk factors also play essential roles in breast cancer development, including the most critical genes as *BRCA*1/2 (5). Despite that only 5–6% of breast cancer cases are inherited, genetic mutations are among the most predictable risk factors contributing to breast cancer development (6).

Analyses of gene expression profile and genome-wide sequencing have demonstrated that the canonical Wnt/β-catenin pathway is involved in processes of breast cancer initiation, proliferation, and metastasis (7). The Wnt signaling is a highly conserved pathway that regulates cellular processes including cell fate determination, organ development, polarity, motility, normal adult homeostasis, stem cell renewal and cancer (8, 9). Activation of Wnt pathway target genes depends on the recruitment of β-catenin into the nucleus. The interaction of TCF/LEF with the β-catenin protein results in the activation of a transcription complex that regulates the expression of target genes participating in tumorigenesis, such as *CCND1*, *PPARD*, *ATOH1*, *CD44*, *FGF20*, *JAG1*, *LGR5*, *MYC*, and *SNAI1* (9–11). Among genes participating in this signaling pathway, *AXIN2* and *TCF7L2* have shown an association with different cancers. The *AXIN2* gene is located on the long arm of chromosome 17q24 and encodes to the Axin2 protein, which participates as a negative regulator of the Wnt/β-catenin pathway, exhibiting a crucial role in cell differentiation, migration, and apoptosis (12). The transcription factor 7-like 2 (*TCF7L2*) gene is located on chromosome 10q25.2, encodes a high mobility group (HMG) box-containing transcription factor involved in cell differentiation and migration (13). Therefore, mutations or variations in the Wnt signaling pathway components triggers the expression of numerous target genes involved in cellular proliferation, evasion of apoptosis, tissue invasion, and metastasis (13, 14).

Several studies have investigated the association of the *AXIN2* and *TCF7L2* polymorphisms with cancer. The *AXIN2* polymorphisms have been associated with colorectal (15, 16), lung (17, 18), prostate (19), and breast cancer (20–23). Specifically, the *AXIN2* rs2240308 (C > T) variant has been assessed and associated with several cancers (15–20); for breast cancer, this variant have been studied only in two different populations, although with inconsistent results (20, 22). For the variants rs7903146 (C > T) and rs12255372 (G > T) of the *TCF7L2* gene, various studies have been conducted in colorectal (24–28), prostate (29), lung (30), and breast cancer (31–34). In breast cancer, these variants show contradictory results among different populations.

We hypothesis that the distribution of alleles, genotypes, and haplotypes of the rs1133683 and rs2240308 variants of the *AXIN2*, and the rs7903146 and rs12255372 variants of the *TCF7L2*, are linked to major clinicopathological characteristics of breast cancer.

## Materials and Method

A total of 404 women were recruited, 202 patients with clinical and histological diagnosis of breast cancer, according to the criteria of the UMAE Gynecology Hospital of the Social Security Mexican Institute (IMSS) in Guadalajara, Mexico. Breast cancer was stratified according to the tumor-node-metastasis (TNM) classification. The control group was 202 unrelated healthy women age-matched with the patient group. The study was approved by the Ethical Committee 1305 (R-2018-1305-004) of West Biomedical Research Center, IMSS, and conducted according to national and international ethical standards. All the participants signed informed consent for participation in this study. A standard epidemiological questionnaire allowed us to collect personal data, including age, gender, drinking and smoking status, familial history, and pharmacological therapy. Women with a previous cancer history were excluded from the control group, while those who had undergone chemotherapy or radiotherapy were excluded from the patient group. Information about the clinical and pathological features of the patients was obtained from the hospital records.

Genomic DNA was extracted from peripheral blood using standard methods (35). The polymorphisms (c.148C>T) rs2240308 and (c.1386C>T) rs1133683 in the *AXIN2* gene and rs7903146 (C > T) and rs12255372 (G > T) in the *TCF7L2* gene were genotyped by polymerase chain reaction-restriction fragment length polymorphism (PCR-RFLP) using the primer pairs described (18, 36). PCR for the rs2240308 polymorphism was performed through 30 cycles in a 10-μL volume containing 100 ng DNA, 10X buffer (500 mM KCl, 100 mM Tris- HCl, and 0.1% Triton TMX-100), 2.0 mM MgCl2, 200 μM dNTPs, 1pM of each primer, and 2 U Taq DNA Polymerase. Denaturation was carried out at 94°C, annealing at 60°C, and elongation at 72°C for 1 min each. Five microliters of the PCR product were digested with 3U of the NsiI restriction enzyme. The digested products were separated on 6% polyacrylamide gels. Fragment observed by electrophoresis corresponded to 218 and 24 bp for the wild-type allele (C) and 242 bp for the polymorphic allele (T). The rs1133683 polymorphism was identified under the same PCR conditions mentioned above. 4U of the restriction enzyme TaqI were used to digest 5 µL of PCR products. The digested products were separated by electrophoresis on 8% polyacrylamide gels. Fragments of 294 bp identify the wild-type allele (C), while fragments of 274 and 20 bp correspond to the polymorphic allele (T). Quality control for these assays was assessed in randomly selected samples that were re-genotyped by an independent technician. The concordance among genotype assays was 100%.

Allele and genotype frequencies were estimated by direct counting in both groups. The chi-square test evaluated the Hardy-Weinberg equilibrium (HWE), as well as the differences in the distribution of alleles and genotypes, and the clinical characteristics between patients and controls. To measure the association of breast cancer with the presence of alleles or genotypes, we made stratified analysis by age, smoking status, TNM stage, and histologic-molecular subtype; also, we calculated the odds ratio (OR) and corresponding 95% confidence intervals (CIs) using SPSS v17.0 software package (SPSS, Inc., Chicago, IL, United States). For all statistical analyzes, *p* < 0.05 was considered significant. A Bonferroni correction test was applied to adjust the *p* values (*p* < 0.012 was considered significant). Interaction of the genetic polymorphisms in both loci of each gene was evaluated using the combined effect and the haplotypes analysis. The linkage disequilibrium and the haplotype frequencies were calculated using the Haploview 4.2 software.

## Results

Table 1 shows the clinicopathological characteristics of breast cancer patients. The mean (SD) age observed was 47.9 ± S.D. 7.37 for the breast cancer group and 46.6 ± S.D. 11.7 years in the control group (*p* = 0.1). Results adjusted by the Bonferroni test (0.012) shown that tobacco consumption was marginally associated in the breast cancer group (*p* = 0.017), while alcohol consumption was not associated with breast cancer (*p* = 0.879).

**TABLE 1:** Clinicopathological data of breast cancer patients.

Characteristics	Breast cancer group *n* = 202 (%)
Body Mass Index (BMI) mean	29.1 (±5.6)
Menopause status	
Pre-menopause	51 (25.2)
Menopause	151 (74.8)
Breastfeeding	
No	44 (21.8)
Yes	
<6 months	30 (14.8)
>6 months	128 (63.4)
Hysterectomy	
Yes	42 (20.8)
No	160 (79.2)
TNM stage	
I	10 (4.9)
II	61 (30.2)
III	59 (29.2)
IV	72 (35.7)
Tumor location	
Unilateral	193 (95.5)
Left	112 (55.5)
Right	81 (40.0)
Bilateral	9 (4.5)
Histology (adenocarcinoma)	
Ductal	176 (87.1)
Lobular	24 (11.9)
Mixed	2 (1.0)
Molecular subtype	
Luminal A	108 (53.5)
Luminal B	51 (25.2)
Her2	28 (13.9)
Triple-negative	15 (7.4)
Metastatic node status	
Positive	155 (76.7)
Negative	47 (23.3)
Metastasis	
Yes	76 (37.6)
No	126 (62.4)

The four analyzed SNPs in the control group were in Hardy-Weinberg equilibrium (all *p* > 0.05). Comparative analysis of the *AXIN2* and *TCF7L2* variants in breast cancer patients and the control group showed significant differences (Table 2). Four different haplotypes were observed in the *AXIN2* and *TCF7L2* genes, but only the T-T haplotype of the *TCF7L2* variants shown statistically significant differences.

**TABLE 2:** Genotype, allele and haplotype frequencies of the *AXIN2* and *TCF7L2* polymorphisms in the breast cancer patients and control group. Numbers in bold represent statistically significant values.

Genotype	Controls *n* = 202 (%)	Breast cancer *n* = 202 (%)	OR (95% CI)	*p* value
***AXIN*2 (rs1133683)**				
C/C	47 (23.3)	45 (22.3)	1.00 (Reference)	
C/T	108 (53.4)	108 (53.4)	1.04 (0.64–1.70)	0.960
T/T	47 (23.3)	49 (24.3)	1.08 (0.61–1.92)	0.884
C/T + T/T vs. C/C	155 (76.7)	157 (77.8)	1.05 (0.66–1.68)	0.905
Allele: C	202 (50.0)	198 (49.0)	1.00 (Reference)	
T	202 (50.0)	206 (51.0)	1.04 (0.78–1.37)	0.832
**AXIN2 (rs2240308)**				
C/C	74 (36.6)	38 (18.8)	1.00 (Reference)	
C/T	103 (51.0)	112 (55.4)	**2.11 (1.31–3.40)**	**0.002**
T/T	25 (12.4)	52 (25.8)	**4.05 (2.18–7.50)**	**0.001**
C/T + T/T vs. C/C	128 (63.4)	164 (81.2)	**2.49 (1.58–3.93)**	**0.001**
Allele: C	251 (62.1)	188 (46.5)	1.00 (Reference)	
T	153 (37.9)	216 (53.5)	**1.88 (1.42–2.49)**	**0.001**
**TCF7L2 (rs7903146)**				
C/C	91 (45.1)	62 (30.7)	1.00 (Reference)	
C/T	94 (46.5)	119 (58.9)	**1.85 (1.21–2.83)**	**0.005**
T/T	17 (8.4)	21 (10.4)	1.81 (0.88–3.71)	0.144
C/T + T/T vs. C/C	111 (54.9)	140 (69.3)	**1.85 (1.23–2.78)**	**0.004**
Allele: C	276 (68.3)	243 (60.1)	1.00 (Reference)	
T	128 (31.7)	161 (39.9)	1.42 (1.06–1.90)	0.018
**TCF7L2 (rs12255372)**				
G/G	119 (58.9)	92 (45.5)	1.00 (Reference)	
G/T	71 (35.1)	78 (38.6)	1.42 (0.93–2.16)	0.125
T/T	12 (6.0)	32 (15.9)	**3.44 (1.68–7.06)**	**0.001**
G/T + T/T vs. G/G	83 (41.0)	110 (54.4)	**1.71 (1.15–2.54)**	**0.009**
Allele: G	309 (76.4)	262 (64.9)	1.00 (Reference)	
T	95 (23.6)	142 (35.1)	**1.76 (1.29–2.39)**	**0.001**
Haplotype				
**AXIN2 rs1133683-rs2240308**				
C-C	69 (34.2)	50 (24.8)	0.63 (0.41–0.97)	0.049
T-T	44 (21.8)	59 (29.2)	1.48 (0.94–2.32)	0.110
T-C	57 (28.2)	44 (21.8)	0.70 (0.45–1.11)	0.167
C-T	32 (15.8)	49 (24.2)	1.70 (1.03–2.79)	0.046
**TCF7L2 rs7903146-rs12255372**				
C-G	110 (54.4)	95 (47.0)	0.74 (0.50–1.09)	0.163
T-G	44 (21.8)	36 (17.8)	0.77 (0.47–1.27)	0.382
T-T	20 (10.0)	44 (21.8)	**2.53 (1.43–4.48)**	**0.001**
C-T	28 (13.8)	27 (13.4)	0.95 (0.54–1.69)	1.000

*p* values were adjusted by the Bonferroni test (0.012).

The results of demographic, and clinicopathological characteristics for each SNP are shown in Tables 3, 4. Concerning the *AXIN2* variants, we did not observe significant differences for the rs1133683 variant when comparing to age, tobacco consumption, TNM stage and histologic molecular subtypes (Table 3). Regarding the *TCF7L2* variants, we observed significant differences when comparing the demographic and clinicopathological characteristics between the breast cancer group and the control group (Table 4).

**TABLE 3:** Association of *AXIN2* genotypes with clinical characteristics of breast cancer. Numbers in bold represent statistically significant values.

	Control/Breast cancer			OR (95% CI); *p* value		
Variable	CC	CT	TT	CT versus CC	TT versus CC	CT + TT versus CC
***AXIN2* rs1133683**						
Age (years)						
<50	24/20	67/57	28/24	1.02 (0.51–2.03); 1.000	1.02 (0.45–2.30); 1.000	1.02 (0.52–1.98); 1.000
≥50	23/25	41/51	19/25	1.14 (0.56–2.30); 0.842	1.21 (0.53–2.75); 0.805	1.16 (0.60–2.25); 0.774
Smoker	8/13	14/26	8/11	1.14 (0.38–3.41); 1.000	0.84 (0.23–3.00); 1.000	1.03 (0.37–2.88); 1.000
TNM Stage						
Stage I + II	47/15	108/38	47/17	1.10 (0.55–2.19); 0.917	1.13 (0.50–2.53); 0.919	1.11 (0.57–2.14); 0.880
Stage III + IV	47/30	108/70	47/32	1.01 (0.58–1.75); 1.000	1.06 (0.56–2.02); 0.973	1.03 (0.61–1.73); 1.000
Stage IV	47/16	108/36	47/20	0.97 (0.49–1.93); 1.000	1.25 (0.57–2.70); 0.710	1.06 (0.55–2.02); 0.985
Histologic molecular subtypes						
Luminal A	47/27	108/53	47/28	0.85 (0.48–1.52); 0.698	1.03 (0.53–2.01); 1.000	0.90 (0.52–1.56); 0.840
Luminal B	47/12	108/27	47/12	0.97 (0.45–2.09); 1.000	1.00 (0.40–2.45); 1.000	0.98 (0.47–2.03); 1.000
Her2	47/3	108/19	47/6	2.75 (0.77–9.76); 0.169	2.00 (0.47–8.47); 0.544	2.62 (0.73–8.74); 0.205
Triple Negative	47/3	108/9	47/3	1.30 (0.33–5.04); 0.951	1.00 (0.19–5.21); 1.000	1.21 (0.32–4.47); 1.000
***AXIN2* rs2240308**						
Age (years)						
<50	43/20	63/58	13/23	1.97 (1.04–3.75); 0.051	**3.80 (1.60–9.01); 0.003**	2.00 (1.07–3.73); 0.038
≥50	31/18	40/54	12/29	2.32 (1.14–4.73); 0.029	**4.16 (1.71–10.12); 0.002**	**2.74 (1.39–5.40); 0.004**
Smoker	15/11	11/29	4/10	3.59 (1.26–10.19); 0.028	3.40 (0.84–13.77); 0.153	3.54 (1.33–9.44); 0.019
TNM Stage						
Stage I + II	74/13	103/37	25/20	2.04 (1.01–4.11); 0.062	**4.55 (1.98–10.47); 0.001**	**2.53 (1.30–4.93); 0.008**
Stage III + IV	74/25	103/75	25/32	**2.15 (1.25–3.70); 0.007**	**3.78 (1.89–7.57); 0.001**	**2.47 (1.46–4.16); 0.001**
Stage IV	74/11	103/38	25/23	2.48 (1.19–5.17); 0.020	**6.18 (2.64–14.47); 0.001**	**3.20 (1.58–6.47); 0.001**
Histologic molecular subtypes						
Luminal A	74/25	103/55	25/28	1.58 (0.90–2.76); 0.141	**3.31 (1.63–6.70); 0.001**	1.91 (1.12–3.26); 0.021
Luminal B	74/7	103/35	25/9	**3.59 (1.51–8.52); 0.004**	3.80 (1.28–11.28); 0.026	**3.63 (1.55–8.47); 0.003**
Her2	74/2	103/16	25/10	5.74 (1.28–25.85); 0.021	**14.8 (3.03–72.17); 0.001**	**10.4 (2.43–44.46); 0.001**
Triple Negative	74/4	103/6	25/5	1.07 (0.29–3.95); 1.000	3.70 (0.92–14.86); 0.120	1.58 (0.48–5.17); 0.618

p values were adjusted by the Bonferroni test (0.012).

**TABLE 4:** Association of *TCF7L2* genotypes with clinical characteristics of breast cancer. Numbers in bold represent statistically significant values.

***TCF7L2*** **(rs7903146)**						
	**Control/Breast cancer**			**OR (95% CI);** ***p*** **value**		
**Characteristic**	**CC**	**CT**	**TT**	**CT versus CC**	**TT versus CC**	**CT + TT versus CC**
Age (years)						
<50	56/28	53/61	10/12	**2.30 (1.28–4.12); 0.007**	2.40 (0.92–6.23); 0.114	**2.31 (1.31–4.07); 0.005**
≥50	35/34	41/58	7/9	1.45 (0.78–2.70); 0.300	1.32 (0.44–3.95); 0.821	1.43 (0.78–2.61); 0.301
Smoker	11/10	17/33	2/7	2.13 (0.75–6.02); 0.237	3.85 (0.64–23.05); 0.260	2.31 (0.83–6.39); 0.168
TNM Stage						
Stage I + II	91/21	94/41	17/8	1.89 (1.03–3.44); 0.051	2.03 (0.77–5.35); 0.231	1.91 (1.06–3.42); 0.039
Stage III + IV	91/41	94/78	17/13	1.84 (1.14–2.96); 0.015	1.69 (0.75–3.81); 0.283	1.81 (1.14–2.88); 0.04
Stage IV	91/20	94/45	17/7	2.17 (1.19–3.97); 0.015	1.87 (0.68–5.11); 0.338	2.13 (1.18–3.82); 0.015
Histologic molecular subtypes						
Luminal A	91/36	94/64	17/8	1.72 (1.04–2.83); 0.044	1.18 (0.47–2.99); 0.898	1.63 (1.00–2.66); 0.060
Luminal B	91/13	94/31	17/7	2.30 (1.13–4.69); 0.028	2.88 (1.00–8.27); 0.086	2.39 (1.20–4.76); 0.017
Her2	91/7	94/17	17/4	2.35 (0.93–5.93); 0.102	3.05 (0.80–11.60); 0.195	2.45 (1.00–6.04); 0.070
Triple Negative	91/6	94/7	17/2	1.12 (0.36–3.48); 1.000	1.78 (0.33–9.59); 0.851	1.22 (0.42–3.58); 0.912
***TCF7L2*** **(rs12255372)**						
	**Control/Breast cancer**			**OR (95% CI);** ***p*** **value**		
**Variable**	**GG**	**GT**	**TT**	**GT versus GG**	**TT versus GG**	**GT + TT versus GG**
Age (years)						
<50	77/75	35/42	7/14	2.05 (1.14–3.66); 0.021	3.42 (1.28–9.10); 0.020	**2.28 (1.32–3.92); 0.004**
≥50	42/47	36/36	5/18	0.89 (0.47–1.66); 0.844	3.21 (1.09–9.42); 0.049	1.17 (0.65–2.10); 0.688
Smoker	21/20	8/22	1/8	2.88 (1.04–7.96); 0.066	8.40 (0.96–73.36); 0.068	3.50 (1.33–9.18); 0.017
TNM Stage						
Stage I + II	119/36	71/25	12/9	1.16 (0.64–2.09); 0.723	2.47 (0.96–6.35); 0.095	1.35 (0.78–2.33); 0.342
Stage III + IV	119/56	71/53	12/23	1.58 (0.98–2.55); 0.075	**4.07 (1.89–8.76); 0.001**	**1.94 (1.24–3.03); 0.004**
Stage IV	119/27	71/34	12/11	2.11 (1.17–3.78); 0.017	**4.04 (1.61–10.12); 0.004**	**2.38 (1.37–4.15); 0.002**
Histologic molecular subtypes						
Luminal A	119/53	71/43	12/12	1.35 (0.82–2.23); 0.278	2.24 (0.94–5.32); 0.101	1.48 (0.92–2.38); 0.123
Luminal B	119/17	71/20	12/14	1.97 (0.96–4.01); 0.087	**8.16 (3.24–20.56); 0.001**	**2.86 (1.50–5.47); 0.001**
Her2	119/15	71/8	12/5	0.89 (0.36–2.21); 0.988	3.30 (1.02–10.68); 0.087	1.24 (0.56–2.74); 0.739
Triple Negative	119/7	71/7	12/1	1.67 (0.56–4.97); 0.513	1.41 (0.16–12.50); 1.000	1.63 (0.57–4.69); 0.511

p values were adjusted by the Bonferroni test (0.012).

## Discussion

It has been described that genetic alterations in Wnt pathway components, such as APC, β-catenin, AXIN2, and TCF7L2 are implicated in colorectal, melanoma, gastric, hepatocellular, and breast cancer (7, 8, 10–12, 37). Some reports have shown increased levels of nuclear or cytoplasmic β-catenin in breast cancer, suggesting activation of the Wnt signaling pathway due to alternative mechanisms such as autocrine signaling or decreased expression of soluble extracellular inhibitors of the Wnt ligand (38–40).

*AXIN2* and *TCF7L2* are polymorphic genes, and several variants have been identified in their exonic and intronic regions. By the first time, we analyzed genotypes and haplotypes of four SNPs located in genes whose protein products participate in the Wnt/β-catenin signaling pathway: rs1133683 (C/T) and rs2240308 (C/T) of the *AXIN2* gene and rs7903146 (C/T) and rs12255372 (G/T) of the *TCF7L2* gene; the results achieved here suggest that the *AXIN2* rs2240308 and *TCF7L2* rs7903146 and rs12255372 variants play a significant role in promoting breast cancer.

As regards *AXIN2* variants, the rs2240308 SNP (c.148 C > T) is a C > T nucleotide change at position 148 of the *AXIN2* exon 1, which produces a substitution of proline by serine at codon 50. The variant rs2240308 (p.Pro50Ser) is located near the RGS domain (regulator of G protein signalling; amino acids 81–200), which involves the APC-binding site and participates in the tumor suppressor function of AXIN2 through the assembly of the β-catenin destruction complex (17–20). We did not find a statistical association of the rs1133683 variant with the demographic and clinicopathological features in these patients. In contrast, patients carrying the C/T or T/T genotypes of the *AXIN2* rs2240308 variant present an increased association regarding age, advanced TNM stages, and positive Her2 subtype of the patients. Wang et al. analyzed the rs2240308 SNP in peripheral blood mononuclear cells of breast cancer patients from North America (22), but no association was found. On the contrary, our results and those described by Aristazabal-Pachon et al. (20) showed that patients who were carriers of the T allele (CT or TT genotypes) had a greater tendency to develop breast cancer. In addition, an association of the CT or TT genotypes of the rs2240308 variant was found with some clinicopathological variables such as TNM stage and histological-molecular subtype. Interestingly, Wang et al. examined the *AXIN2* rs4791171 variant and reported a significant risk in younger patients with breast cancer (22); meanwhile, Alanazi et al. examined the *AXIN2* rs3923086 variant and found a significant protective association for breast cancer in younger patients who also had the negative estrogen receptor subtype (21). To our knowledge, this is the second study reporting the association of the rs2240308 variant with breast cancer risk. Neither association was found with the haplotypes of these polymorphisms.

As regards *TCF7L2* variants, several studies have shown an association between some *TCF7L2* variants with different types of cancer (24, 26, 29–32, 41), which is explained because the TCF4 protein, involved in the Wnt/β-catenin signaling pathway, acts as a transcription factor that induces expression of some oncogenes as *CCND1* and *MYC*, involved in proliferation, apoptosis, invasion, and metastasis (9, 40). It has been described that the most frequent *TCF7L2* variants (rs7903146, intron 3 and rs12255372, intron 4) affect the mRNA stability and the alternative splicing (24, 26). Specifically, in breast cancer, these *TCF7L2* variants (rs12255372 and rs7903146) shown results contradictories regards to cancer susceptibility (21, 29, 31–34, 41). In this study, we found an association of the genotypes CT and TT of both variants with breast cancer risk. Patients carrying the C/T heterozygous genotype of the variant rs7903146 showed a significant risk to develop breast cancer, including in younger patients (<50 years). Likewise, patients carrying the homozygous T/T genotype of the *TCF7L2* rs12255372 variant showed a significant association with breast cancer and with the possibility of reach the advanced TNM stages III + IV. These patients also exhibited an increased risk to develop luminal B tumors. The haplotype analysis found that patients carrying the T-T haplotype (rs7903146 and rs12255372 SNPs) show increased breast cancer frequency. In brief, our results found a higher cancer risk for women carrying the CT genotype compared to women carrying the GG genotype of the *TCF7L2* 7903146 variant. This association was also statistically significant for patients under 50 years of age and with tumors in advanced TNM stages (III + IV). The frequencies discrepancies observed between our study and other studies could be related to population differences, ethnic diversity, sample size, and different histologically tumors.

In conclusion, our study shows that the C/T and T/T genotypes of the *AXIN2* rs2240308 variant and the *TCF7L2* rs7903146 C/T and rs12255372 T/T genotypes are linked to breast cancer. Furthermore, some genotypes are also significantly associated with advanced stages of TNM, the histology-molecular subtypes of breast cancer, and the age of the patients. Additional studies, including larger samples and functional analysis of the polymorphisms, are necessary to confirm and extend our findings. We speculate that these gene variants may be important in the pathogenesis and/or susceptibility to this disease.

## Summary Table

### What Is Known About This Subject

• Mutations or genetic polymorphisms in the *TCF7L2* and *AXIN2* genes have been associated with different cancers.
• Axin2 protein participates as a negative regulator of the Wnt/β-catenin pathway with a crucial role in cell differentiation, migration, and apoptosis.
• The transcription factor 7-like 2 is involved in cell differentiation and migration.

### What This Paper Adds

• Frequencies of the CT and TT genotypes of the *AXIN2* rs2240308 variant are significantly higher in breast cancer patients. These same genotypes were associated with advanced TNM stages and histologic molecular subtypes in breast cancer patients.
• Frequencies of the CT or GT and TT genotypes of the *TCF7L2* rs7903146 and rs12255372 variants are significantly higher in breast cancer patients. These genotypes were also associated with age, advanced TNM stages and histologic molecular subtypes in breast cancer patients.

## Data Availability

The original contributions presented in the study are included in the article/supplementary material, further inquiries can be directed to the corresponding author.
